# Micronization Effects on Structural, Functional, and Antioxidant Properties of Wheat Bran

**DOI:** 10.3390/foods12010098

**Published:** 2022-12-25

**Authors:** Sitong Lai, Zhenjia Chen, Yanqing Zhang, Guang Li, Yuanmeng Wang, Qingliang Cui

**Affiliations:** 1 College of Agricultural Engineering, Shanxi Agricultural University, Jinzhong 030801, China; 2 College of Food Science and Engineering, Shanxi Agricultural University, Jinzhong 030801, China

**Keywords:** micronization, particle size, wheat bran, structural properties, functional properties, antioxidant properties

## Abstract

To explore the effect of micronization on the structural, functional, and antioxidant properties of wheat bran, wheat bran with mean particle size (*D*_50_) of 46.08, 34.29, 26.51, 26.35, and 26.05 μm were prepared by using an ultrafine pulverizer under different rolling frequencies (0, 6, 9, 12, and 15 times). The main chemical components and structural, functional, and antioxidant properties of the wheat bran were compared and a correlation analysis was conducted. As the *D*_50_ of the wheat bran decreased from 46.08 μm to 26.05 μm, the micromorphology exhibited the destruction of the bundle structure, which is formed by starch and fiber, during which the starch particles peeled off, the fiber fragments destructed, and some of the slim fiber fragments attached to the surfaces of the starch granules. According to the X-ray diffraction pattern, part of the crystalline structure was transformed into an amorphous structure and the crystallization index decreased from 13.08% to 3.95%. According to the near-infrared spectrum, more active groups, such as the hydroxyl group, were exposed; however, no new groups were generated. These structural changes accordingly caused changes in the chemical components, functional properties, and antioxidant properties of the wheat bran. Specifically, the protein, total phenols, total flavonoids, and fatty acid content increased by 6.72%, 23.47%, 19.07%, and 172.88%, respectively. The lipase activity, antioxidant activity in vitro (DPPH• scavenging activity, ABTS^+^• scavenging activity, and ferric reducing antioxidant power), and the water-holding, cholesterol-adsorption, sodium nitrite-adsorption, and cation-exchange capacities, were enhanced to some extent. The oil-holding capacity decreased from 3.01 g/g to 1.32 g/g. The swelling capacity decreased first and then increased and the swelling capacity of the wheat bran with a *D*_50_ of 34.29 μm was the lowest (3.62 mL/g). Therefore, the micronization could be used as a pretreatment method to improve the functional and antioxidant properties of wheat bran; however, the optimal particle size of wheat bran is based on the function of the product.

## 1. Introduction

Wheat (*Triticum eastivum* L.) is widely cultivated worldwide and widely utilized in the production of flour, malt [1], beer [2], and a range of bakery products [3]. Wheat bran is a by-product of refined wheat flour production, accounting for 20–30% of the total wheat quality. Compared with refined wheat flour, wheat bran is rich in protein, dietary fiber, vitamins, minerals, phenols, and other nutrients. These nutrients regulate intestinal flora, promote intestinal peristalsis, absorb harmful substances, and prevent cardiovascular diseases [4,5,6]. However, wheat bran is often used as animal feed due to its high fiber content, inferior palatability, and sensory morphology, which have limited its application in food [7]. For example, wheat bran breaks the continuous and uniform dough structure and confers the unpopular color, volume, and texture of products [8].

Researchers have verified the use of micronization as one of the effective means of improving the sensory quality of high-fiber food [9]. According to the existing research, reducing the particle size of highland barley bran improved the taste, caking degree, and reconstitution stability of the products [9]. Reducing the particle size of rice flour produced a dense structure in sweet dumplings, which exhibited a strong water-retention capacity and low cooking-loss rate [10]. Properly reducing the particle size of wheat bran substantially improved the textural and cooking properties of noodles, which led a high sensory score [11]. Bread created from coarse whole wheat flour had a less compact structure and softer texture [12]. Thus, decreasing the particle size of wheat bran could increase its compatibility with the food market. Researchers have used moderate micronization to enhance the functional properties of raw materials. For instance, Ma et al. [13] found that when they reduced the particle size of cumin dietary fiber from 380 μm to 120 μm, the water-holding, swelling, and fat-absorption capacities increased. However, these properties decreased when the particle size of the cumin dietary fiber was decreased to 106 μm. Liang et al. [14] reported that fine millet bran powder exhibited a higher protein content, soluble dietary fiber content, total phenolic content, and antioxidant capacity, but a lower water-holding capacity, than its coarse counterpart. With the decrease in the particle size of unripe banana flour, the total phenolic content and antioxidant activity first increased and then decreased and the particles of 212–315 μm exhibited higher total phenolic contents and better antioxidant activities than those of the other particle sizes [15]. Based on these findings, the functional and antioxidant properties of different materials may exhibit different trends with the decreases in their particle sizes. 

Wheat bran is a by-product with high nutritional value and high yields. The researchers demonstrated that micronization improved the consumer acceptance of whole wheat noodles and bread [11,12]. However, there are few comprehensive and systematic reports on the micronization effects on the functional and antioxidant properties of wheat bran. Therefore, to expand the application of wheat bran in the food market, the functional and antioxidant properties of wheat bran by micronization were determined. Furthermore, the structural properties of wheat bran to explain the changes in functional and antioxidant properties were explored.

## 2. Materials and Methods

### 2.1. Materials

The Ronghe Flour Processing Factory (Yuncheng, Shanxi, China) supplied the wheat bran. The wheat variety was Jinmai 73 and the flour extraction rate was 70%.

### 2.2. Preparation of Wheat Bran

The wheat bran was obtained using an ultra-micro-pulverizer (ZKY-303, Zhong Ke Hao Yu Technology Development Co. Ltd., Beijing, China) under different rolling frequencies (6, 9, 12, and 15 times) and at a constant time (30 min) and was recorded as B1, B2, B3, and B4, respectively. The wheat bran directly obtained from the factory was recorded as B0. 

### 2.3. Particle Size Distribution

The particle size distribution of the wheat bran was measured in the wet dispersion mode using ethanol and ultrasonication by a laser particle size analyzer (2600E, Bettersize Instrument Ltd., Dandong, China). The refraction index was 1.53 and the absorption parameter was 0.7 [16]. The results were expressed as *D*_10_, *D*_50_, and *D*_90_, which represented the corresponding particle sizes when the cumulative particle size distribution reached 10%, 50%, and 90%, respectively. 

With reference to Zhang [17], the span of the wheat bran was calculated using Equation (1):

(1) $$\mathrm{Span}=\frac{D_{90}-D_{10}}{D_{50}}$$

### 2.4. Main Chemical Components

The water, ash, crude protein, and crude fat content were determined according to the GB 5009-2016 method [18,19,20,21]. The total flavonoids content was determined according to the DB 43/T476-2009 method [22]. The total phenols content was determined according to the method of Blagoj et al. [23]. The results were calculated by the dry weight.

### 2.5. Scanning Electron Microscopy (SEM)

An appropriate amount of wheat bran was evenly spread on the conductive film and sprayed with gold in a vacuum environment. Then, the microscopic morphology of the wheat bran was observed using a scanning electron microscope (JSM–7500F, JJEOL Co., Ltd., Tokyo, Japan) at magnifications of 200, 500, and 1000 times. The length of a single starch particle was measured using the ruler tool of Adobe Photoshop (Adobe Photoshop CC2017).

### 2.6. X-ray Diffraction (XRD) 

The crystalline structure of the wheat bran was measured using an X-ray diffractometer (D/Max2550VB/PC, Rigaku Corporation, Tokyo, Japan), according to the method of Zhang et al. [24]. 

An appropriate amount of wheat bran was pressed into a sheet and then the sheet was scanned with Cu-Kα radial under 40 kV and 30 mA and at a diffraction angle (2*θ*) that ranged from 5° to 40° at 2°/min in steps of 0.02°. Then, the crystallinity index (CI) of the wheat bran was calculated using Equation (2):

(2) $$\mathrm{Crystallinity}\;\mathrm{index}\;\left(\%\right)=\frac{I_{002}-I_{am}}{I_{002}}\times 100\;$$

where *I*_002_ is the maximum intensity of the diffraction angle and *I_am_* is the diffraction intensity at 2*θ* = 18°.

### 2.7. Fourier-Transform Infrared Spectroscopy (FTIR)

The spectra were obtained using a near-infrared spectrometer (Spectrum Two N, PerkinElmer Co. Ltd., MA, USA). For the measurement, the KBr crystal was first dried and ground. Then, 200 mg of the KBr crystal was mixed with 2 mg of wheat bran and then the mixture was pressed into a sheet. The sheets were scanned 32 times, during which the wave number ranged from 450 to 4000 cm^−1^, at a nominal resolution of 4 cm^−1^. The spectra were analyzed with air as the blanks.

### 2.8. Fatty Acids Value (FAC) 

The fatty acid value of the wheat bran was determined according to the GB/T 15684-2015 method [25]; 5 g of sample was mixed with 30 mL of ethanol and the mixture was shaken at 25 °C for 1 h. After centrifugation at 2000× *g* for 5 min, 20 mL of supernatant was mixed with 5 drops of phenolphthalein solution and then titrated with KOH–ethanol solution. The supernatant was replaced with 20 mL of ethanol and then titrated with the same KOH–ethanol solution, which was used as the blank. The FAC of the wheat bran was calculated using Equation (3):

(3) $$\mathrm{FAC}\left(\mathrm{mg}\;\mathrm{KOH}/100\;g\right)=\frac{(V_{1}-V_{0})\times C\times M_{r}}{m}\times \frac{30}{20}\times \frac{100}{100-w}\times 100$$

where *C* is the concentration of the KOH–ethanol solution (mol/L); *V*_1_ is the volume of KOH–ethanol solution that the supernatant consumed (mL); *V*_0_ is the volume of KOH–ethanol solution that the blank consumed (mL); M_r_ is the molar mass of the KOH (g/mol); *m* is the weight of the sample (g); and *w* is the moisture content of the wheat bran (%).

### 2.9. Lipase Activity (LA) 

The lipase activity of the wheat bran was determined according to the GB/T 5523-2008 method [26]; 2 g of sample was mixed with 1 mL of oil and then 5 mL of phosphate buffer (pH 7.4, 0.05 mol/L) and 4 mL of deionized water were added. The mixture was incubated for 24 h at 30 °C. After that, 50 mL of ethanol–ethyl acetate solution with a mixing ratio of 4:1 (V:V) was added. After complete mixing, the mixture was sat for 5 min before filtering. Then, 25 mL of the filtrate was mixed with 5 drops of phenolphthalein solution and titrated with the KOH–ethanol solution. In addition, the above operation with 2 g of wheat bran was repeated except for the one incubating for 24 h, which was used as the blank. The lipase activity of the wheat bran was calculated using Equation (4):

(4) $$\mathrm{LA}\;\left(\mathrm{mg}\;\mathrm{KOH}/g\right)=\frac{(V_{1}-V_{0})\times C\times M_{r}}{m}\times \frac{60}{25}\times \frac{100}{100-w}$$

where *C* is the concentration of KOH–ethanol solution (mol/L); *V*_1_ is the volume of KOH–ethanol solution that the filtrate consumed (mL); *V*_0_ is the volume of KOH–ethanol solution that the blank consumed (mL); M_r_ is the molar mass of the KOH (g/mol); *m* is the weight of the sample (g); and *w* is the moisture content of the wheat bran (%).

### 2.10. Lipoxygenase (LOX) Activity 

The lipoxygenase activity of the wheat bran was determined according to the method described by Cato et al. [27], with slight modifications. First, to prepare the linoleic acid substrate, 0.5 mL of Tween 20 was mixed with 10 mL boric acid buffer solution (pH 9.0, 0.05 mol/L) and 0.5 mL of linoleic acid was slowly added. Then, 1.3 mL of sodium hydroxide solution (1 mol/L) was slowly added and the mixture was mixed until clear. Then, the mixture was diluted to 200 mL with a boric acid buffer solution (pH 9.0, 0.05 mol/L) and the pH was adjusted to 7.0 with HCl solution (1.0 mol/L). Finally, 0.3 mL of the above mixture was mixed with 9.5 mL of sodium acetate buffer solution (pH 5.6, 0.05 mol/L), which was referred as the linoleic acid substrate; 0.4 g of the sample was mixed with 10 mL of phosphate-buffered solution (pH 7.5, 0.05 mol/L). After incubation for 30 min at 4 ℃, the mixture was centrifugated at 8000 rpm for 10 min at 4℃. After, 20 μL of supernatant was added to 3 mL of the linoleic acid substrate and a linear absorbance curve at 234 nm within 3 min was obtained. The Δabs/g·min^−1^ was the LOX activity unit.

### 2.11. Water-Holding Capacity (WHC)

The water-holding capacity of the wheat bran was determined according to the method described by Jiang et al. [28], with slight modification; 0.5 g of the sample was mixed with 10 mL of distilled water in a centrifuge tube (15 mL). The mixture was shaken at 37 °C for 4 h and then centrifugated at 4000 rpm for 15 min. After discarding the supernatant, the remaining mixture was weighed. The WHC of the wheat bran was calculated using Equation (5):

(5) $$\mathrm{WHC}\;\left(g/g\right)=\frac{m_{2}-m_{0}}{m_{1}-m_{0}}\times \frac{100}{100-w}$$

where *m*_0_ is the weight of the centrifuge tube (g); *m*_1_ is the initial weight of the centrifuge tube and sample (g); *m*_2_ is the weight of the centrifuge tube and precipitate (g); and *w* is the moisture content of the sample (%).

### 2.12. Oil-Holding Capacity (OHC)

The oil-holding capacity of the wheat bran was determined according to the method described by Jiang et al. [28], with slight modification; 0.5 g of the sample was mixed with 4 g of oil in a centrifuge tube (15 mL). The mixture was shaken at 37 °C for 4 h and then centrifugated at 4000 rpm for 15 min. After discarding the supernatant, the remaining mixture was weighed. The OHC of the wheat bran was calculated using Equation (6):

(6) $$\mathrm{OHC}\;\left(g/g\right)=\frac{m_{2}-m_{0}}{m_{1}-m_{0}}\times \frac{100}{100-w}$$

where *m*_0_ is the weight of the centrifuge tube (g); *m*_1_ is the initial weight of the centrifuge tube and sample (g); *m*_2_ is the weight of the centrifuge tube and precipitate (g); and *w* is the moisture content of the sample (%).

### 2.13. Swelling Capacity (SC)

The swelling capacity of the wheat bran was determined according to the method described by Jiang et al. [28], with slight modification; 0.5 g of sample and 8 mL of distilled water was mixed in a 10 mL measuring cylinder and the volume of the suspension was recorded. The cylinder was sealed with plastic wrap and incubated at 25 °C for 24 h. After that, the volume of the suspension was recorded. The SC of the wheat bran was calculated using Equation (7):

(7) $$\mathrm{SC}\;\left(\mathrm{mL}/g\right)=\frac{V_{1}-V_{0}}{m}\times \frac{100}{100-w}\;$$

where *V*_0_ is the initial volume of the suspension (mL); *V*_1_ is the volume of the suspension after incubation (mL); *m* is the weight of the sample (g); and *w* is the moisture content of the sample (%).

### 2.14. Cholesterol-Adsorption Capacity (CAC) 

The cholesterol-adsorption capacity of the wheat bran was determined according to the method described by Zhu et al. [29], with slight modification; 5 mL of cholesterol standard solution (acetic acid was the solvent) with the concentrations of 0, 0.1, 0.2, 0.3, 0.4, and 0.5 mg/mL were mixed with 15 mL of O-phthalaldehyde solution (0.1 mg/mL; acetic acid was the solvent) and 10 mL of 98.3% sulfuric acid (*m*/*m*). The mixture was sat for 10 min after full mixing and then the absorbance value was measured at 550 nm. Then, the standard curve equation of the cholesterol concentration and absorbance value was plotted [18]. In this study, the equation was *A* = 0.0537*C* − 0.0221 (*R^2^* = 0.9991), where *A* represents the absorbance value and *C* represents the cholesterol solution concentration (μg/mL). 

To prepare the cholesterol latex, 15 g of fresh egg yolk liquid was mixed with 500 mL of distilled water and the cholesterol latex concentration was determined. Then, 0.2 g of the wheat bran was mixed with 10 mL of the above cholesterol latex. The pH of the mixture was adjusted to 2.0 or 7.0 and the mixture was shaken at 37 °C for 2 h. The cholesterol concentration of the supernatant after centrifugation (4000 rpm, 20 min) was determined. The CAC of the wheat bran was calculated using Equation (8):

(8) $$\mathrm{CAC}\;\left(\mathrm{mg}/g\right)=\frac{\left(C_{0}-C_{1}\right)\times V}{m}\times \frac{100}{100-w}$$

where *C*_0_ is the initial concentration of the cholesterol latex (mg/mL); *C*_1_ is the cholesterol concentration of the supernatant (mg/mL); *V* is the volume of the cholesterol latex added in the system; *m* is the weight of the sample (g); and *w* is the moisture content of the sample (%).

### 2.15. Sodium Nitrite-Adsorption Capacity (SNAC)

The sodium nitrite-adsorption capacity of the wheat bran was determined according to the GB 5009.33-2016 method [30]. First, the standard curve equation of the sodium nitrite concentration and absorbance value was plotted. In this study, the equation was *A* = 0.7393*C* + 0.001 (*R*^2^ = 0.9985), where *A* represents the absorbance value and *C* represents the concentration of the sodium nitrite solution (μg/mL). 

First, 0.1 g of wheat bran was mixed with 10 mL of the sodium nitrite standard solution (5.0 μg/mL). The pH of the mixture was adjusted to 2.0 or 7.0 and then the mixture was shaken at 37 °C for 2 h. The sodium nitrite concentration of the supernatant after centrifugation (4000 rpm, 20 min) was determined. The SNAC of the wheat bran was calculated using Equation (9):

(9) $$\mathrm{SNAC}\;\left(\mu g/g\right)=\frac{\left(C_{1}-C_{0}\right)\times V}{m}\times \frac{100}{100-w}$$

where *C*_1_ is the sodium nitrite concentration of the supernatant (μg/mL); *C*_0_ is the concentration of the sodium nitrite standard solution (μg/mL); *V* is the volume of the standard solution added in the system (μg/mL); *m* is the weight of the sample (g); and *w* is the moisture content of the sample (%).

### 2.16. Cation-Exchange Capacity (CEC)

The cation-exchange capacity of the wheat bran was determined according to the method described by Ma et al. [13], with slight modification; 1.0 g of wheat bran was mixed with 50 mL of hydrochloric acid solution (0.1 mol/L). The mixture was sat at 25 °C for 12 h. After that, the mixture was filtered and washed with distilled water until the scrubbing solution was presented as neutral and free of chloride ions, which was identified by pH paper and 10% (*m*/*m*) AgNO_3_ solution. After drying at 55 °C, 0.25 g of residue was mixed with 100 mL of 15% NaCl solution (*m*/*V*) and stirred for 30 min. Then, the pH of the mixture was recorded each time after adding 0.2 mL of 0.1 mol/L NaOH solution, until the pH steadily changed. Finally, the pH curve of the mixture change with the volume of the added NaOH solution was plotted. 

### 2.17. DPPH• Scavenging Capacity 

The DPPH• scavenging capacity of the wheat bran was determined according to the method described by Liang et al. [14], with slight modification. To prepare the extract, the sample was mixed with 70% ethanol (*V*/*V*) at 1:50 (*m*/*V*, dry basis) and the extraction was performed under ultrasonic conditions (180 w) at 60 °C for 30 min. Then, after centrifugation (4000 rpm, 20 min), the supernatant was used for the determination of the DPPH• scavenging capacity, ABTS^+^• scavenging capacity, and ferric-reducing antioxidant power.

Next, 0.2 mL of the extract was added to 3.8 mL of the DPPH• solution (0.02 mmol/L). The mixture was reacted at 25 °C in the dark for 30 min and then the absorbance value was measured at 517 nm (*A*_1_). The absorbance value of the mixture was also measured, which replaced the extract with 70% ethanol or DPPH• solution with ethanol, at 517 nm (*A*_0_ and *A*_2_, respectively). The DPPH• scavenging capacity of the wheat bran was calculated using Equation (10):

(10) $$\mathrm{Scavenging}\;\mathrm{capacity}\;\mathrm{for}\;\mathrm{DPPH}•\left(\%\right)=\left[1-\frac{\left(A_{1}-A_{2}\right)}{A_{0}}\right]\times 100$$

The standard curve equation of the VC concentration (10–80 μg/mL) and absorbance value by replacing the extract with the VC solution was plotted. In this study, the equation was *A* = 0.8381*C* − 5.3732 (*R*^2^ = 0.9951), where *A* represents the absorbance value and *C* represents the concentration of the VC solution (μg/mL). Additionally, the VC equivalent (mg/g) was calculated.

### 2.18. ABTS^+^• Scavenging Capacity 

The ABTS^+^• scavenging capacity of the wheat bran was determined according to the method described by Aprotosoaie et al. [31], with slight modification. To prepare the ABTS^+^• solution, 5 mL of ABTS solution (7 mmol/L) and the equivalent volume of potassium persulfate solution (2.45 mmol/L) were mixed and reacted for 12–16 h in the dark. After that, the mixture was diluted to an absorption value of 0.700 ± 0.003 at a wavelength of 734 nm. 

Next, 0.1 mL of the extract was added to 3.9 mL of ABTS^+^• solution. After being reacted at 25 °C in the dark for 15 min, the absorbance value of the mixture was measured at 734 nm (*A*_1_). The absorbance value of the mixture was also measured, which replaced the extract with 70% ethanol or ABTS^+^• solution with ethanol at 517 nm (*A*_0_ and *A*_2_, respectively). The ABTS^+^• scavenging capacity of the wheat bran was calculated using Equation (11):

(11) $${\mathrm{Scavenging}\;\mathrm{capacity}\;\mathrm{for}\;\mathrm{ABTS}}^{+}•\left(\%\right)=\left[1-\frac{\left(A_{1}-A_{2}\right)}{A_{0}}\right]\times 100$$

The standard curve equation of the VC concentration (20–140 μg/mL) and absorbance value by replacing the extract with the VC solution was plotted. In this study, the equation was *A* = 0.6111*C* − 1.4558 (*R*^2^ = 0.9963), where *A* represents the absorbance value and *C* represents the concentration of the VC solution (μg/mL). Additionally, the VC equivalent (mg/g) was calculated.

### 2.19. Ferric-Reducing Antioxidant Power (FRAP)

The ferric-reducing antioxidant power of the wheat bran was determined according to the method described by the references, with slight modification [32,33]. To prepare the TPTZ work solution, 20 mmol/L FeCl_3_ solution, 10 mmol/L TPTZ solution (40 mmol/L; HCl was the solvent), and 300 mmol/L CH_3_COONa buffer solution (pH: 3.6) at 1:1:10 (*V*:*V*:*V*) were mixed and prepared for use.

To plot the standard curve, 0, 25, 50, 100, 150, and 200 μL of the FeSO_4_ standard solution (1 mmol/L) were mixed with the quantitative distilled water to the total volume to 200 μL; 4 mL of TPTZ working solution was added and reacted for 10 min at 37 °C, then the absorbance value was measured at 593 nm. Then, the standard curve equation of the FeSO_4_ concentration (μmol/L) and absorbance value was plotted. In this study, the equation was *A* = 0.0200*C* − 0.0175 (*R*^2^ = 0.9972), where *A* represents the absorbance value and *C* represents the FeSO_4_ solution concentration. 

Next, 0.2 mL of the extract was added to 4 mL of the TPTZ working solution, reacted at 37 °C for 10 min, and then the absorbance value was measured at 593 nm. According to the standard curve, the concentration of the FeSO4 in the solution after the reaction was calculated. The FRAP of the wheat bran was calculated using Equation (12):

(12) $$\mathrm{FRAP}\;\left(\mu \mathrm{mol}/g\right)=\frac{C_{1}\times V_{1}}{C_{0}\times V_{0}}\times \frac{1}{1000}$$

where *C*_1_ is the concentration of the FeSO4 in solution after reaction (μmol/L); *V*_1_ is the volume of the reaction system (mL); *C*_0_ is the concentration of the extract (g/mL); and *V*_0_ is the volume of the extract (mL).

### 2.20. Statistical Analysis

All the experiments were performed in triplicate and the results were present as means ± standard errors. The Duncan’s test was used to analyze the variance (ANOVA) and the difference was considered significant at *p <* 0.05 with the software SPSS17.0 for Windows. The Pearson’s correlation analysis was performed with the software Origin version 2017 for Windows.

## 3. Results and Discussion

### 3.1. Particle Size Distribution

**Figure 1:**
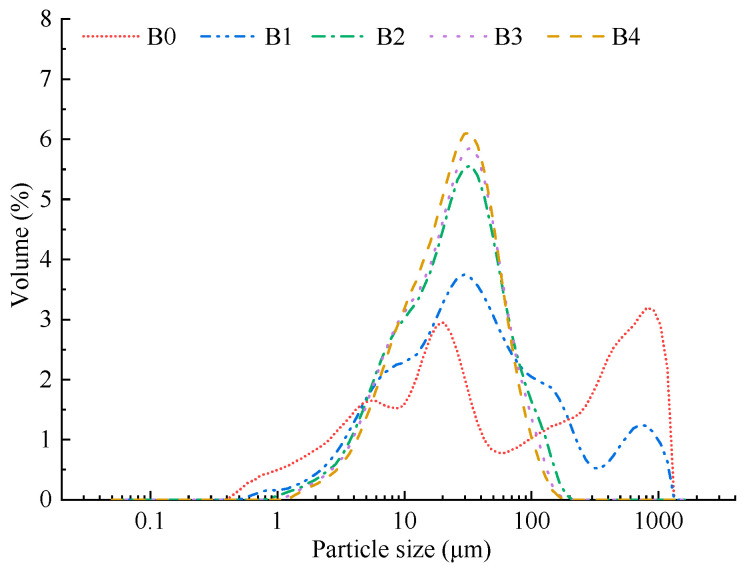
Particle size distribution patterns of the wheat bran. The B0, B1, B2, B3, and B4 are the wheat bran prepared under the rolling frequencies 0, 6, 9, 12, and 15 times/s *D_10_*, *D_50_*, and *D_90_* are mean particle sizes at 10%, 50%, and 90% of the volume distribution, respectively.

The particle size distribution directly reflects the micronization effect. The particle size distribution patterns of the wheat bran prepared under different rolling frequencies are presented in Figure 1. The particle size distributions of B0 and B1 ranged from 0.50 μm to 173.00 μm and those of B2, B3, and B4 ranged from 1.00 μm to 186.20 μm. These values indicated that a rolling frequency of nine times substantially reduced the particle size of the wheat bran, when the milling time was 30 min. With the increase in the rolling frequency, the wheat bran gradually changed from a multipeak distribution to a bimodal distribution and finally presented a high and narrow unimodal distribution. The change suggested that the size distribution of the wheat bran was more concentrated and the size was more uniform, which was consistent with the results of the span (Table 1). With the increase in the rolling frequency from 6 times to 15 times, the *D*_50_ of the wheat bran substantially decreased from 46.08 μm to 26.05 μm, which could be because of the high-strength rolling that was highly destructive to the wheat bran structure, as Cappelli et al. [34] have verified. Zhang et al. [17] reported that the *D*_50_ of the tobacco leaf flour presented strong correlations with the water-holding capacity, oil-holding capacity, and antioxidant activity. Therefore, the functional properties of micronized wheat bran were further investigated in this study.

### 3.2. Main Chemical Components

The main chemical components of the micronized wheat bran are presented in Table 2. The protein content of the wheat bran was 15.36–15.86%, which was substantially higher than those of corn flour and highland barley bran [35,36]. With the decrease in the *D*_50_ of the wheat bran, the water content decreased from 9.95% to 5.59%, which corresponded to the finding obtained by Jin et al. [37]. The decrease could be ascribed to the intense mechanical force on the wheat bran during the rolling process, which contributed to the temperature increase in the crusher cavity. The high-intensity rolling leads to a more obvious increasing temperature, so that the water in the material more easily evaporates [34]. At the same time, with the decrease in the *D*_50_ of the wheat bran, the dissolved amounts of fat, protein, total flavonoids, and total phenols in the wheat bran increased by 20.73%, 6.72%, 23.47%, and 19.07%, respectively. The particles with smaller sizes had higher specific surface areas and more damage structure, which promoted the release of the nutrients in their original bound states [38]. However, Bala et al. [39] found that when the *D*_50_ of pea flour decreased from 249 μm to 74 μm, the dissolved amount of protein demonstrated an adverse trend. The two opposite trends may be a result of the different preparation methods of the powder. The pea protein may be denatured during the crushing process due to its special protein structure. Micronization improves the dissolution of the active components, which provides guidance for the development of functional food. 

### 3.3. Surface Micromorphology

**Figure 2:**
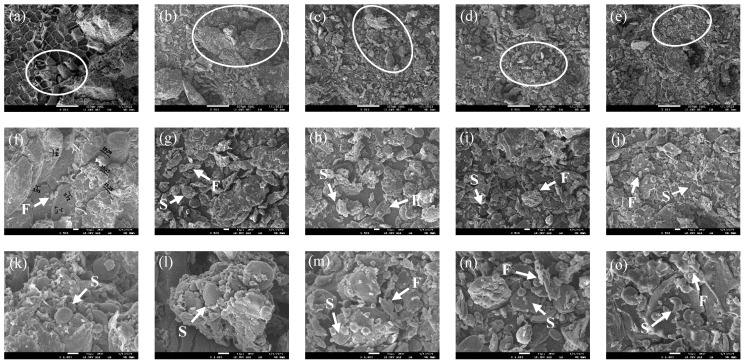
Surface micromorphology of the wheat bran. The magnifications of (**a**–**e**) (B0, B1, B2, B3, and B4) (**f**–**j**) (B0, B1, B2, B3, and B4), and (**k**–**o**) (B0, B1, B2, B3, and B4) are 200×, 500×, and 1000×, respectively. F refers to fiber fragments and S refers to starch granules. The B0, B1, B2, B3, and B4 are the wheat bran prepared under the rolling frequencies 0, 6, 9, 12, and 15 times.

To observe the micromorphology of the wheat bran, the surface is magnified by 200, 500, and 1000 times using a scanning electron microscope and the results are presented in Figure 2. The inner surface of the bran contained many starch clusters and most of the starch particles were embedded in an ordered structure; however, some of the starch particles were exposed (Figure 2a,f,k). The particle size of the wheat bran substantially decreased and the particle size distribution was more uniform, which was consistent with the result of the particle size distribution (Figure 2a–e). The complete ellipsoidal and spherical scattered starch granules can be observed in Figure 2g, as well as the bundle structure formed by starch and fiber. With the increase in the *D*_50_, the bundle structure was destroyed by mechanical action, the starch particles peeled off, and the fibers presented irregular fragments (Figure 2h–j,m–o). In addition, the degree of fiber fragmentation increased and some small fragments were attached to the surfaces of the starch particles, which is similar to the observations of Wang et al. [9] on highland barley bran. The damage starch particle can be especially observed in Figure 2o, which may have been damaged by the strong mechanical action.

The lengths of the starch granules ranged from 3.91 µm to 19.09 µm, which is in accordance with the measurements reported by Singh et al. [40]. According to the results of the B0 particle size distribution, the particle distribution with particle sizes <10 µm was mainly caused by the starch particles and the particle distribution with particle sizes >100 µm was mainly caused by the bundle structure that was formed by the fiber and starch particles. With the increase in the rolling frequency, the bundle structure was destroyed by mechanical action and the degree of the fiber fragmentation increased, which led to an increased specific surface area. The increasing specific surface area promoted electrostatic action, causing the adsorption of the starch particles on the surface of the fiber fragments and formation of new combinations of starch and fiber. Finally, the particle size distribution of the wheat bran was close to the middle, resulting in a high and narrow single-peak distribution. These changes in the wheat bran structure could affect the stacking mode between the particles, which could result in changes in the functional properties.

### 3.4. Crystalline Structure 

**Figure 3:**
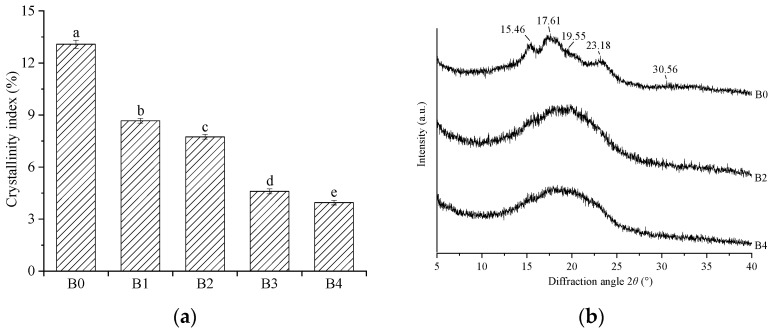
Crystallinity index (**a**), X-ray diffraction pattern (**b**), and the wheat bran. The B0, B1, B2, B3, and B4 are the wheat bran prepared under the rolling frequencies 0, 6, 9, 12, and 15 times. Different lowercase letters on the shoulders of the column indicate significant differences (*p* < 0.05).

The crystal structure of the wheat bran is characterized using X-ray diffraction and the crystalline index (CI) was presented in Figure 3a. With the decrease in the *D*_50_ of the wheat bran, the CI significantly decreased from 13.08% to 3.95% (*p* < 0.05). The reduction in the CI might be explained by the crystalline region that is converted into amorphous cellulose and soluble sugar, which is caused by the destruction of the hydrogen bonds in the crystalline regions of cellulose and hemicellulose after strong mechanical action [41]. In previous studies, researchers demonstrated that the CI decreased with the decrease in the particle sizes of black tea powder [24]. The diffraction patterns of B0, B2, and B4 (Figure 3b) were further compared. The crystalline diffraction peaks of cellulose and hemicellulose usually occur in a 2*θ* range from 15° to 25° [41]. In B0, the obvious diffraction peaks at 15.46°, 17.61°, 19.55°, and 23.18° and weak diffraction peaks at 30.56° were observed, which suggests that it has a typical cellulose I-type structure in which the crystalline regions coexist with amorphous regions [36]. However, only a broad and deep diffraction peak at a 2*θ* of 20° was observed in B2. Compared with B2, B4 presented a wider and shallower diffraction peak. The diffraction patterns were in accordance with the results of the crystallinity index. The decrease in the CI indicates that wheat bran presents a looser structure, which is conducive to contact with other substances and improved its functional properties.

### 3.5. Functional Group

**Figure 4:**
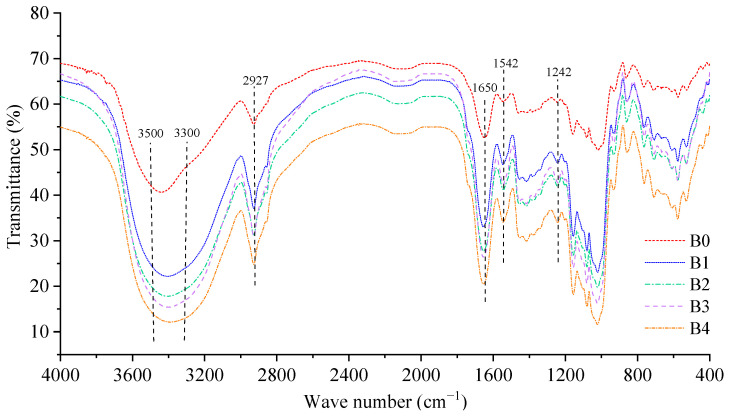
Infrared spectrum scan of the wheat bran. The B0, B1, B2, B3, and B4 are the wheat bran prepared under the rolling frequencies 0, 6, 9, 12, and 15 times.

Fourier-transform near-infrared spectroscopy (FTIR) is used to characterize the functional groups of the wheat bran and the results are presented in Figure 4. The absorption peak at 2927 cm^−1^ was caused by the stretching vibration of C–H in CH_2_ or CH_3_ from the polysaccharides [41]. The absorption peak at 1542 cm^−1^ marked the amide II-absorption band of the secondary amide group, indicating that the wheat bran contained protein [36]. A weak absorption peak at 1242 cm^−1^ corresponded to the stretching vibrations of the carbonyl and benzene rings and acetyl groups from lipids [36]. According to these absorption peaks, the wheat bran contained polysaccharides, proteins, lipids, and other components, which is in agreement with the chemical compound results. The strong and broad absorption peak at 3300–3500 cm^−1^ was caused by the O–H stretching vibration within or between the cellulose and hemicellulose molecules [41]. With the increase in the rolling frequency, the peak shape of the absorption peak was wider and stronger, indicating that more hydroxyl groups were exposed, which might have resulted from the broken glycosidic bonds. The decrease in the CI of the wheat bran also contributed to the exposure of more hydroxyl groups due to the fact that the amorphous cellulose exhibited more free hydroxyl groups. In general, with the decrease in the particle size of the wheat bran, the shapes of the infrared spectra and positions of the absorption peaks were similar; however, the transmittance decreased. According to these results, no new functional groups were generated; however, the number of functional groups increased during the micronization, which is consistent with the results of Ma and Zhao et al. [13,36]. Therefore, micronization is a physical modification method that could be used to promote the exposure of more functional groups in wheat bran.

### 3.6. Fatty Acid Value (FAV)

**Figure 5:**
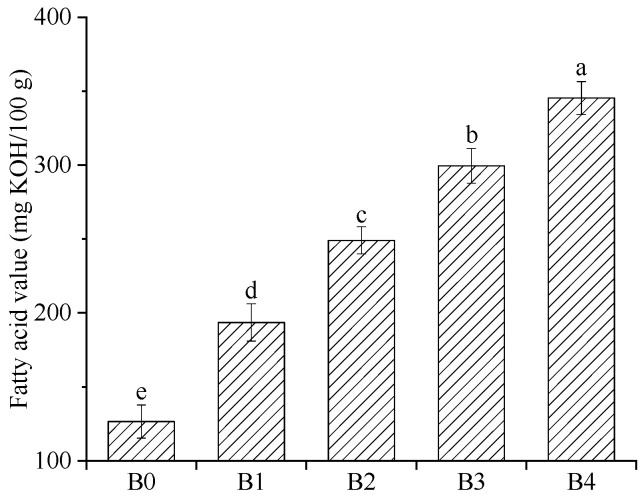
Fatty acid value of the wheat bran. The B0, B1, B2, B3, and B4 are the wheat bran prepared under the rolling frequencies 0, 6, 9, 12, and 15 times. Different lowercase letters on the shoulders of the column indicate significant differences (*p* < 0.05).

Lipid oxidation is a particular problem in wheat bran; it leads to an off flavor [42]. The fatty acid value is used to reflect the degree of lipid hydrolysis and the lipolysis is the first step of lipid oxidation [42]. The fatty acid value of the wheat bran is evaluated and the results are presented in Figure 5. With the increase in the *D*_50_, the FAC of the wheat bran substantially increased from 126.56 to 345.36 mg KOH/100 g (*p* < 0.05), indicating that the decrease in the particle size of the wheat bran accelerated the lipid hydrolysis reaction, which may be because the high rolling frequency increased the fat dissolution of the wheat bran, as shown in Table 2. At the same time, the high rolling frequency promoted the contact between the lipase and lipid, which resulted in the acceleration of the lipid hydrolysis reaction. If the wheat bran is mixed with refined wheat flour at a ratio of 3:7 (m/m) as whole wheat flour, then the FAV of the whole wheat flour would be between 37.97 and 103.61 mg KOH/100 g, although the FAV of the refined wheat flour is ignored. According to the standard [43], the FAV of the whole wheat flour should not exceed 116.00 mg KOH/100 g. Thus, some measures should be considered to control the increase in the fatty acid value in storage, especially for wheat bran with small particle sizes.

### 3.7. Lipase and Lipoxygenase Activities 

**Figure 6:**
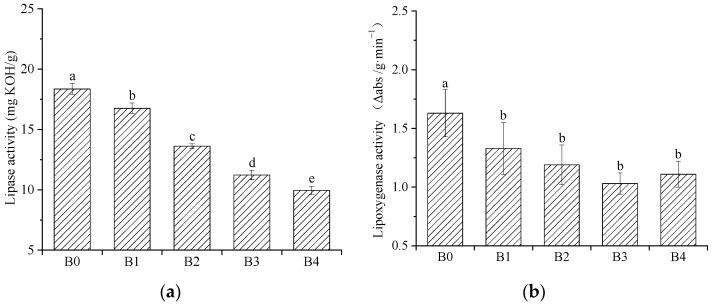
Lipase activity (**a**) and lipoxygenase activity (**b**) of wheat bran. The B0, B1, B2, B3, and B4 are the wheat bran prepared under the rolling frequency 0, 6, 9, 12, and 15 times. Different lowercase letters on the shoulders of the column indicate significant differences (*p* < 0.05).

Lipase generally acts on the ester bond of triglyceride and releases fatty acids [44]. Lipoxygenase catalyzes the oxidation of unsaturated fatty acids, such as linoleic acid and linolenic acid, to generate hydrogen peroxide, which is further cracked into small molecular compounds and results in the inferior flavor and functional properties of food [45]. Thus, the lipase and lipoxygenase activities of the wheat bran are related to the functional properties. The lipase and lipoxygenase activities of the wheat bran are presented in Figure 6. With the increase in the *D*_50_ from 46.08 to 26.05 μm, the lipase activity of the wheat bran decreased 45.75%. The difference in the lipoxygenase activity between B1, B2, B3, and B4 was not substantial; however, it was lower than that of B0. The decreasing lipase activity may have been due to the changes in the enzyme structure [46]. Gao et al. [47] reported that phenolic compounds could interact with lipase via hydrogen bonds to change the spatial structure of pancreatic lipase and inhibit its activity. According to the results, the lipoxygenase had relatively stable structures compared with the lipase, which is consistent with the results of Jia et al. [42]. 

### 3.8. Water-Holding Capacity (WHC), Oil-Holding Capacity (OHC), and Swelling Capacity (SC)

**Figure 7:**
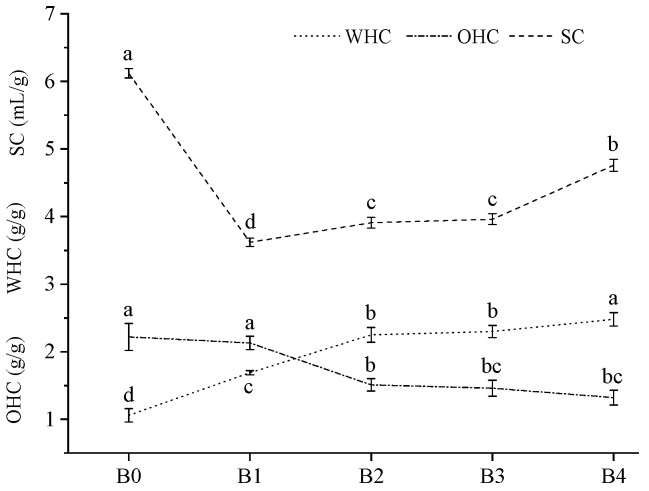
The water-holding capacity (WHC), oil-holding capacity (OHC), and swelling capacity (SC) of the wheat bran. The B0, B1, B2, B3, and B4 are the wheat bran prepared under the rolling frequencies 0, 6, 9, 12, and 15 times. Different lowercase letters on the shoulders of the same indicator indicate significant differences (*p* < 0.05).

The high WHC of wheat bran can promote human defecation, which is conducive to the prevention of the incidence of hemorrhoids and intestinal cancer [36]. The high OHC of wheat bran can reduce the absorption of oil in the intestines and stomach, which can prevent obesity and hyperlipidemia [39]. The high SC of wheat bran provides a sense of satiety for the stomach and intestines, which can also prevent obesity [9]. The WHC, OHC, and SC of the wheat bran are evaluated and the results are presented in Figure 7. With the increase in the *D*_50_, the WHC of the wheat bran increased from 1.06 g/g to 2.48 g/g and the OHC decreased from 3.01 g/g to 1.32 g/g. The SC first increased and then decreased. The SC of B1 was the lowest (3.62 mL/g). Generally, the WHC, OHC, and SC of materials are mainly affected by the accumulation between particles, the pore size, and their binding ability to water molecules [9]. With the decrease in the *D*_50_ of the wheat bran, the pores formed by the accumulation between the particles decreased, which led to the stronger physical retention on the water and oil molecules. The OHC and SC of B1 were lower than those of B0. As the specific surface area of the wheat bran increase, more hydrophilic groups are exposed, which can enhance the binding ability to water. Thus, the WHC and SC of the wheat bran tended to increase when the *D*_50_ was less than 34.29 μm. Based on the above analysis, the OHC was mainly related to the physical retention of the pores between the particles and the WHC was mainly related to the number of hydrophilic groups on the particle surface.

### 3.9. Cholesterol-Absorption Capacity (CAC) and Sodium Nitrite-Absorption Capacity (SNAC)

**Figure 8:**
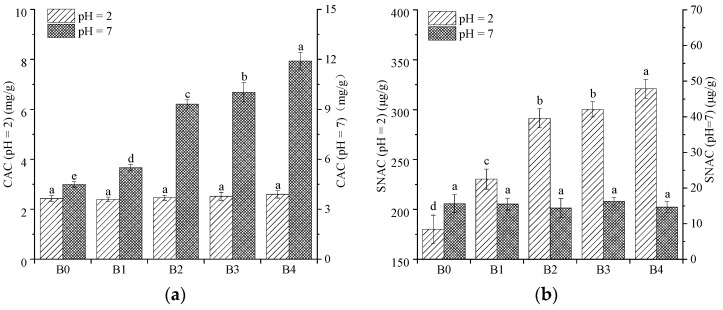
Cholesterol-absorption capacity (CAC, (**a**)) and sodium nitrite-absorption capacity (SNAC, (**b**)) of the wheat bran. The B0, B1, B2, B3, and B4 are the wheat bran prepared under the rolling frequencies 0, 6, 9, 12, and 15 times. Different lowercase letters on the shoulders of the column indicate significant differences (*p* < 0.05).

Dietary fiber can absorb cholesterol, sodium nitrite, lead ion, glucose, and other substances, which can prevent diabetes, cardiovascular disease, colon cancer, and other diseases [41]. Wheat bran contains 35–39% dietary fiber [48]. The CAC and SNAC of the wheat bran are determined and the results are presented in Figure 8. The ACC of the wheat bran in the pH = 7 environment (simulated small intestine) was 2.29–4.58 times more than in the pH = 2 environment (simulated stomach), which indicates that wheat bran has a strong adsorption capacity for cholesterol in the small intestine (Figure 8a). In acidic environments, both cholesterol and dietary fiber present partial positive charges, which result in repulsion that is not conducive to the adsorption of cholesterol. However, the SNAC of the wheat bran in the pH = 2 environment (simulated stomach) was 11.54–21.94 times more than that in the pH = 7 environment (simulated small intestine), which indicates that wheat bran has a strong adsorption capacity for sodium nitrite in the stomach (Figure 8b). In acidic environments, the phenolic acid group of wheat bran reacts with NO_2_^-^ [36] and the compounds that contain carbonyl groups dissociate [13], which contribute to the strong adsorption capacity for sodium nitrite in the stomach. As the *D*_50_ of the wheat bran decreased, the CAC (pH = 7) and SNAC (pH = 2) increased. The small particles had larger specific surface areas, which increased the contact areas with cholesterol and sodium nitrite; this could explain the high CAC and SNAC [24]. The high rolling frequency promoted the conversion of insoluble dietary fiber into soluble dietary fiber, which led to the exposure of more active sites and, thus, the enhancement of the CAC and SNAC [48].

### 3.10. Cation Exchange Capacity (CEC)

**Figure 9:**
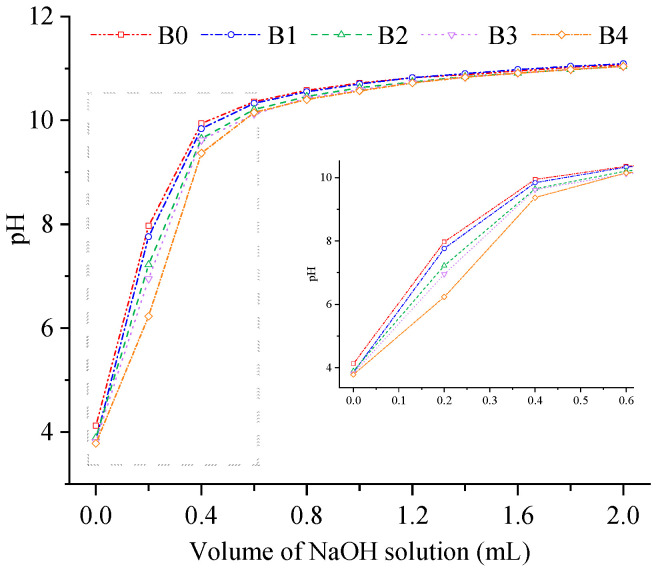
Cation exchange capacity of the wheat bran. The B0, B1, B2, B3, and B4 are the wheat bran prepared under the rolling frequencies 0, 6, 9, 12, and 15 times.

Dietary fiber can exchange with Na^+^ and K^+^ in the intestine, which leads to a decrease in the ratio of Na^+^/K^+^ in the blood and plays a role in lowering blood pressure [36]. Moreover, the hydroxyl, carboxyl, amino, and other side-chain groups in dietary fiber can combine with cations and eliminate them from the body, which results in an instantaneously lower cation concentration in the digestive tract. The low cation concentration can maintain the original environment of the digestive tract and facilitate digestion and absorption. The substances with strong CEC can better maintain the pH value of the solution. Wheat bran is rich in dietary fiber [49]. The CEC of the wheat bran is determined and the results are presented in Figure 9. With the increase in the NaOH solution, the pH value of the solution rapidly increased at first, then slowly increased, and finally tended toward stabilization. When the volume of the added NaOH solution increased from 0 mL to 0.6 mL, B4 could better maintain the pH value of the solution. According to the results, decreasing the particle size of the wheat bran improved the CEC and could provide a stronger buffering environment for the body. Wang et al. [9] obtained similar results from observing the CEC of highland barley bran with different particle sizes. Combined with the infrared spectrum of wheat bran in this study, it is speculated that the high CEC might be due to the exposure of more active groups, which promotes cation exchange. Based on this analysis, B4 can better trap, destroy, and decompose lipids, which prevents the diffusion and absorption of lipids or cholesterol in the human body [48]. 

### 3.11. Antioxidant Activity In Vitro

**Figure 10:**
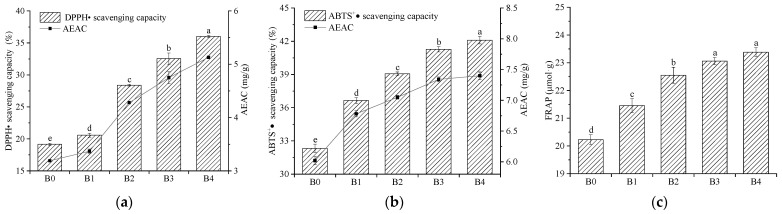
The antioxidant activity of wheat bran in vitro: (**a**) DPPH• scavenging capacity; (**b**) ABTS^+^• scavenging capacity; (**c**) Ferric reducing antioxidant power (FRAP). The B0, B1, B2, B3, and B4 are the wheat bran prepared under the rolling frequencies 0, 6, 9, 12, and 15 times. AEAC is the VC equivalent (mg/g). Different lowercase letters on the shoulders of the column indicate significant differences (*p* < 0.05).

Arteriosclerosis, heart disease, diabetes, liver disease, and other diseases are related to oxygen free radicals. Antioxidant substances can maintain the balance of free radicals and reduce the harm of oxygen free radicals to the body [50]. Chemical groups, such as the phenolic hydroxyl group in flavonoids, provide hydrogen or electrons, and promote the conversion of lipid peroxidation free radicals into relatively stable lipid peroxide, contributing to the elimination of the superoxide anions and other free radicals [50]. Wheat bran contains 2.94–3.63 mg/g of total flavonoids, as demonstrated in this study. The antioxidant activity of wheat bran in vitro is determined and the results are presented in Figure 10. Compared with B0, the DPPH• scavenging capacity, ABTS^+^• scavenging capacity, and ferric reducing antioxidant power (FRAP) of B4 increased by 88.00%, 30.26%, and 15.57%, respectively. The increases indicated that the micronization substantially improved the antioxidant activity of the wheat bran in vitro (*p* < 0.05). Liang et al. [14] verified a similar change, reporting that the DPPH• scavenging capacity and ABTS^+^• scavenging capacity improved with the decrease in the particle sizes of foxtail millet bran powder, which was due to the destruction of the aleurone layer during the micronization that resulted in the release of antioxidant compounds, such as phenolic acids, from the aleurone layer [51]. Moreover, the partial covalent bonds between the ferulic acid and arabinan were broken, which promoted the release of the free phenolic compounds [52]. As the rolling frequency increased, the increasing broken actions for the wheat bran promoted the release of antioxidant compounds, which resulted in an increase in the antioxidant activity [53]. However, the results contrast with those reported by Zhang et al. [54], who indicated that the antioxidant activity of oat bran decreased when the grinding time reached 10 min, which was due to the fact that the *D*_50_ of oat bran was 12.76 μm and was smaller than the particle size of the wheat bran used in this study. According to these results, the particle size affected the antioxidant activity. 

### 3.12. Correlation Analysis 

**Figure 11:**
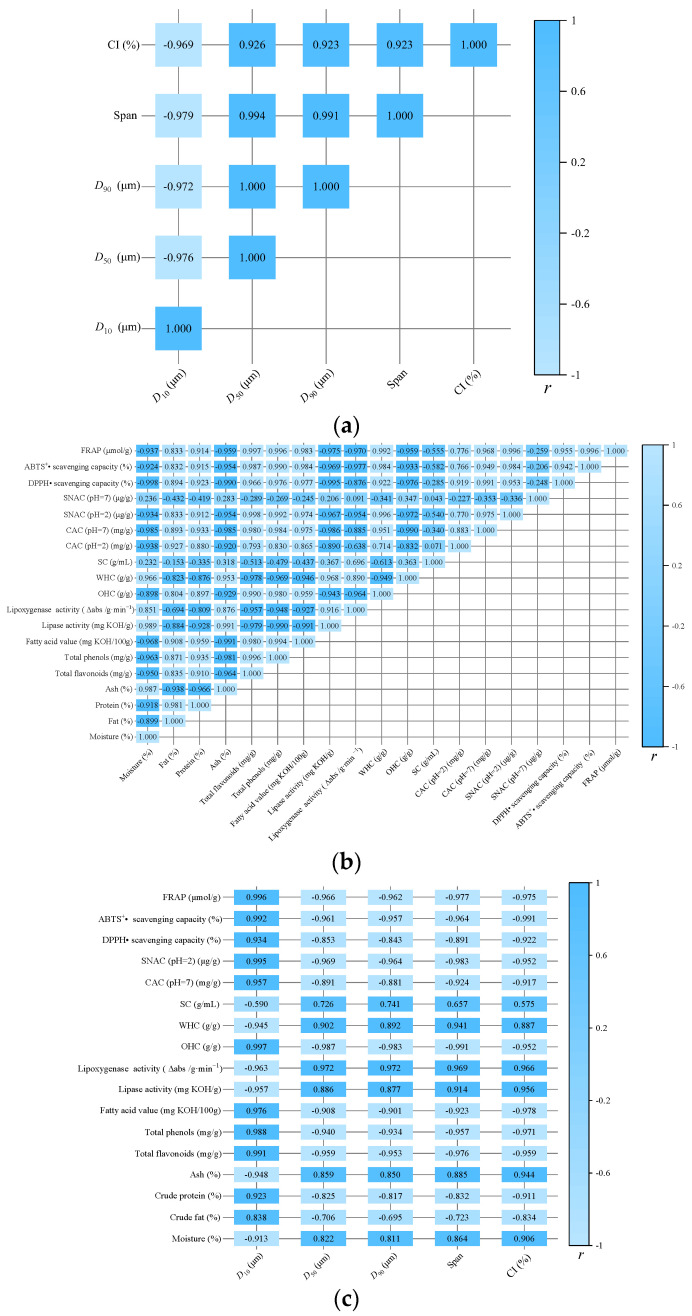
Heatmap of correlation analysis: (**a**) correlation analysis between structural properties; (**b**) correlation analysis among chemical composition, functional properties, and antioxidant properties; and (**c**) correlation analysis between structural properties and chemical composition, functional properties, and antioxidant properties. *r* is the correlation coefficient.

As illustrated in Figure 11a, the crystallinity index (CI) was negatively correlated with the *D*_50_ (*r* = −0.926, *p* < 0.05), *D*_90_ (*r* = −0.923, *p* < 0.05), span (*r* = −0.923, *p* < 0.05), and *D*_10_ (*r* = −0.969, *p* < 0.01), which suggests that the decrease in the particle size of the wheat bran resulted in a lower CI. The total flavonoids were significantly positively correlated with the DPPH• scavenging capacity (*r* = 0.966, *p* < 0.01), ABTS^+^• scavenging capacity (*r* = 0.987, *p* < 0.01), and ferric–reducing antioxidant power (FRAP) (*r* = 0.997, *p* < 0.01). The total phenols were significantly positively correlated with the DPPH• scavenging capacity (*r* = 0.976, *p* < 0.01), ABTS^+^• scavenging capacity (*r* = 0.990, *p* < 0.01), and FRAP (*r* = 0.996, *p* < 0.01) (Figure 11b). Kaur and Li et al. [53,55] reported the strong positive correlation between total flavonoids or total phenols and antioxidant activity and the correlation coefficient of the total phenols and FRAP was higher than that of the DPPH• scavenging capacity, which was due to the lower sensitivity of the DPPH• to hydrophilic antioxidants [55]. As illustrated in Figure 11c, the CI was negatively correlated with the total phenols, total flavonoids, FAV, WHC, CAC, SNAC, and antioxidant activity (*r* < −0.900, *p* < 0.05) and it was positively correlated with the lipase activity (*r* = 0.956, *p* < 0.05), lipoxygenase activity (*r* = 0.966, *p* < 0.05), and OHC (*r* = 0.887, *p* < 0.05). However, the CI was not correlated with the SC (*r* = 0.575, *p* < 0.05). Therefore, the main functional properties of the product need to be considered when the material is micronized. 

## 4. Conclusions

In this study, the effect of micronization on the structural, functional, and antioxidant properties of wheat bran was investigated. According to the results, wheat bran has a typical cellulose I-type structure. With the increase in the rolling frequency from 6 times to 15 times, the starch particles peeled off from the original ordered structure, the fiber fragments were destroyed, the active groups were exposed, and the crystallinity index decreased. Moreover, the *D*_50_ of the wheat bran decreased from 34.29 μm to 26.05 μm. These structural changes prompted the release of protein, total phenols, and total flavonoids. Moreover, the fatty acid value (FAC), water-holding capacity (WHC), cation-exchange capacity (CEC), cholesterol-adsorption capacity (CAC), sodium nitrite-adsorption capacity (SNAC), and antioxidant activity in vitro increased. However, the lipase activity and oil-holding capacity (OHC) decreased and the swelling capacity (SC) first decreased and then increased. According to the correlation analysis, the structural, functional, and antioxidant properties had a strong correlation. It is speculated that the OHC might be primarily affected by the accumulated pores between the particles and that the WHC, CEC, CAC, SNAC, and antioxidant activity might be affected by the number of active groups. In general, micronization can be used as a pretreatment method to improve the functional and antioxidant properties of wheat bran; however, the optimal particle size of wheat bran is based on the function of the product.

## Figures and Tables

**Table 1:** Particle size distribution of wheat bran.

Sample	*D*_10_ (µm)	*D*_50_ (µm)	*D*_90_ (µm)	Span
B0	3.50 ± 0.04 ^e^	46.08 ± 0.09 ^a^	912.70 ± 3.42 ^a^	19.73 ± 0.03 ^a^
B1	5.17 ± 0.04 ^d^	34.29 ± 0.09 ^b^	394.90 ± 3.36 ^b^	11.37 ± 0.09 ^b^
B2	6.39 ± 0.05 ^c^	26.51 ± 0.04 ^c^	76.43 ± 1.40 ^c^	2.64 ± 0.05 ^c^
B3	6.70 ± 0.09 ^b^	26.35 ± 0.03 ^d^	69.49 ± 0.50 ^d^	2.38 ± 0.01 ^d^
B4	7.30 ± 0.11 ^a^	26.05 ± 0.10 ^e^	65.37 ± 1.43 ^d^	2.23 ± 0.04 ^e^

The B0, B1, B2, B3, and B4 are the wheat bran prepared under the rolling frequencies 0, 6, 9, 12, and 15 times. *D*_10_, *D*_50_, and *D*_90_ are mean particle sizes at 10%, 50%, and 90% of the volume distribution, respectively. Different lowercase letters on the shoulders of the same indicator indicate significant differences (*p* < 0.05).

**Table 2:** Main chemical components of the wheat bran.

Component	B0	B1	B2	B3	B4
Moisture (%)	9.95 ± 0.14 ^a^	9.82 ± 0.10 ^a^	7.78 ± 0.11 ^b^	6.58 ± 0.06 ^c^	5.59 ± 0.10 ^d^
Crude Fat (%)	4.92 ± 0.12 ^d^	5.22 ± 0.12 ^c^	5.39 ± 0.11 ^bc^	5.46 ± 0.09 ^b^	5.94 ± 0.10 ^a^
Crude protein (%)	15.36 ± 0.12 ^b^	15.44 ± 0.15 ^b^	15.52 ± 0.15 ^b^	15.56 ± 0.15 ^b^	15.86 ± 0.13 ^a^
Ash (%)	2.93 ± 0.09 ^a^	2.85 ± 0.09 ^a^	2.71 ± 0.05 ^b^	2.62 ± 0.06 ^bc^	2.49 ± 0.10 ^c^
Total flavonoids (mg/g)	2.94 ± 0.04 ^e^	3.16 ± 0.04 ^d^	3.46 ± 0.03 ^c^	3.55 ± 0.04 ^b^	3.63 ± 0.03 ^a^
Total phenols (mg/g)	2.36 ± 0.05 ^e^	2.5 ± 0.04 ^d^	2.66 ± 0.03 ^c^	2.74 ± 0.04 ^b^	2.81 ± 0.03 ^a^

The B0, B1, B2, B3, and B4 are the wheat bran prepared under the rolling frequencies 0, 6, 9, 12, and 15 times. Different lowercase letters on the shoulders of the same indicator indicate significant differences (*p* < 0.05).

## Data Availability

Available upon request from the corresponding author.
